# Transcriptional Profiling of Homologous Recombination Pathway Genes in *Mycobacterium bovis* BCG Moreau

**DOI:** 10.3390/microorganisms11102534

**Published:** 2023-10-11

**Authors:** Marcos Gustavo Araujo Schwarz, Paloma Rezende Corrêa, Leila Mendonça-Lima

**Affiliations:** Laboratório de Biologia Molecular Aplicada à Micobactérias, Instituto Oswaldo Cruz, Fiocruz, Rio de Janeiro 21040-900, RJ, Brazil; pah.rez.correa@gmail.com (P.R.C.); lmlima@ioc.fiocruz.br (L.M.-L.)

**Keywords:** homologous recombination, *Mycobacterium bovis* BCG, DNA damage response pathways, ultraviolet radiation exposure survival

## Abstract

*Mycobacterium bovis* BCG Moreau is the main Brazilian strain for vaccination against tuberculosis. It is considered an early strain, more like the original BCG, whereas BCG Pasteur, largely used as a reference, belongs to the late strain clade. BCG Moreau, contrary to Pasteur, is naturally deficient in homologous recombination (HR). In this work, using a UV exposure test, we aimed to detect differences in the survival of various BCG strains after DNA damage. Transcription of core and regulatory HR genes was further analyzed using RT-qPCR, aiming to identify the molecular agent responsible for this phenotype. We show that early strains share the Moreau low survival rate after UV exposure, whereas late strains mimic the Pasteur phenotype, indicating that this increase in HR efficiency is linked to the evolutionary clade history. Additionally, RT-qPCR shows that BCG Moreau has an overall lower level of these transcripts than Pasteur, indicating a correlation between this gene expression profile and HR efficiency. Further assays should be performed to fully identify the molecular mechanism that may explain this differential phenotype between early and late BCG strains.

## 1. Introduction

Vaccination with *Mycobacterium bovis* BCG is the only available prophylactic method against tuberculosis, a disease that, despite the availability of vaccine and antibiotic therapeutics, still has a high death toll per year, as demonstrated by the 1.6 million deaths related to this pathogen in 2021 [1]. The human tuberculosis etiologic agent is *M. tuberculosis* (*M.tb*), a slow-growing mycobacterium evolutionarily close to *M. bovis*, the cattle pathogen used for the generation of the attenuated *M. bovis* BCG strain first utilized for vaccination in 1921 [2]. This original BCG was distributed worldwide but accumulated specific mutations due to the distinct cultivation methods employed in each location until seed lots and standardized production protocols were put in place [3]. Today, there is a family of different BCG strains with regard to both genomic and phenotypic features [4].

Due to its non-pathogenic profile, BCG is used as a model to study *M.tb*. It is also known that BCG can induce several unspecific immunological responses in humans, helping to protect or improve infection outcomes for other pathogens [5]. This led to an interest in introducing other pathogen antigen genes in BCG to allow its use as a single recombinant vaccine for multiple targets [6,7,8,9]. For this, BCG must undergo genetic manipulations, either to allow reverse genetic approaches to identify a possible important gene by studying its knockout phenotype or by stably introducing foreign genes into its genome. Several genetic tools can be used, such as those based on the homologous recombination (HR) machinery allowing allelic substitution [10,11]. This technique is based on the creation of an allelic exchange substrate (AES), either in a linear form or cloned in a suicide plasmid. After introducing it in the bacterial cell, DNA damage in the target locus will activate HR to exchange this sequence with the one in the AES, thus inserting the desired allele in the proper genomic locus [12]. Usually, markers are used to select the desired legitimate HR events.

We previously showed that, contrary to the BCG Pasteur reference strain, BCG Moreau, the Brazilian strain used for vaccination, is deficient in HR machinery. In BCG Moreau, no knockouts were produced when using HR-based protocols, but only when exogenous mycobacteriophage recombinases coded by the pJV53 plasmid were induced within this strain [13]. The molecular mechanisms underlying this differential phenotype are still not completely known.

Here, we aimed to use survival to ultraviolet (UV) exposure as a diagnostic tool for HR machinery efficiency. It is long known that UV generates DNA damage, usually forming adjacent pyrimidine dimers and slowing down DNA metabolism, such as DNA replication [14,15]. This induces a global SOS response, modulating the expression of several pathways, including the induction of DNA repair mechanisms such as HR [16].

The HR metabolism employs a set of core enzymes involved in three major steps: pre-synaptic (*adnA*, *adnB*); recombination (*recA*); and resolution of Holliday junctions (*ruvA*, *ruvB*, *ruvC*) [17]. Additionally, there are regulatory genes controlling the expression of the HR machinery, both in the SOS response pathway that is LexA/RecA dependent (*lexA*) [16,18] and in the LexA/RecA-independent (*pafB*, *pafC*) pathway [19,20,21,22], where the first represents a repression mechanism and the second an activator one. There is also *recX*, a gene downstream of *recA*, that codes a protein that can inhibit RecA activity by both directly binding to it and to the growing single-strand loaded with the RecA polymer, thus inhibiting single-strand invasion. It is also shown that RecX may have regulatory activity upon *recA* expression [23].

We first standardized the UV exposure assay to detect survival differences between the HR-efficient BCG Pasteur strain and the HR-deficient Moreau strain. The same protocol was then used to evaluate several other BCG strains belonging to different genomic groups (DU1, DU2-IV, DU2-III, DU2-II, and DU2-I) and distinct in vitro evolutionary steps (early [like Moreau] to late [like Pasteur] strains, separated by the RD2 deletion) [24,25]. Furthermore, transcription profiling of the core HR machinery genes and known regulators was used to better understand the molecular events resulting in the different HR phenotypes, comparing BCG Moreau and Pasteur. The resulting data help enlighten the molecular mechanism underlying BCG Moreau HR deficiency.

## 2. Materials and Methods

### 2.1. M. bovis BCG Culture Conditions

*M. bovis* BCG Moreau was provided by Fundação Ataulpho de Paiva (FAP), and the other BCG strains were obtained from the Pasteur Institute’s (Paris, France) culture collection. All *M. bovis* BCG strains were cultivated in 7H9 or 7H10 medium supplemented with a 10% albumin/dextrose/catalase (ADC) mixture at 37 °C. Liquid cultures were started at O.D._600nm_, set to 0.1, and maintained under agitation (200 rpm).

### 2.2. UV Irradiation Assay

We adapted a protocol previously used for BCG Pasteur [26]. Briefly, bacteria from a 200 mL culture (O.D._600nm_~1.0) were collected by centrifugation at 1.000× *g* for 10 min at room temperature in two equally divided tubes, one for the non-irradiated control and the other for the test. Each bacterial pellet was resuspended in 10 mL of the recovered medium after centrifugation. The suspension was then evenly spread onto a 90 mm diameter glass Petri dish.

The assay was conducted under a standard germicidal UV lamp, maintaining a distance of 20 cm between the lamp and the Petri dish. To assess the optimal condition for detecting differences between the HR-deficient and control bacteria, BCG Moreau and Pasteur strains, respectively, were irradiated for different periods (30, 60, 90, and 120 s). A non-irradiated control sample was included for each strain, in which the Petri dish lid was added before irradiation. The total culture volume of each sample was restored to 100 mL with the recovered medium, and the cultivation conditions were maintained for 24 h. This step was added to enhance HR gene induction as a *recA* peak was previously observed under this condition for *M.tb* [27].

Following this restoration period, aliquots were taken from each sample and appropriately diluted before plating onto 7H10 + 10% ADC medium. The plates were incubated at 37 °C for three weeks and CFU values were determined by counting the colonies on each plate.

To determine the UV exposure survival rate profile within the BCG clade, the same assay was performed using different BCG strains (Phipps, Frappier, Connaught, Glaxo, Danish, Prague, Sweden, Japan, and Russia). However, only the 30 s irradiation time was used for this experiment. The survival rate (%) was calculated as (100 × CFU_irradiated_)/CFU_control_. Mean values and standard deviations were calculated from three independent experiments.

### 2.3. RNA Extraction and RT-qPCR

RT-qPCR assays were performed to compare the transcription levels of some of the LexA/RecA dependent (*lexA*, *recA* and *recX*), independent (*pafB*, *pafC*, *adnA* and *adnB*), and mixed (*ruvC*, *ruvA* and *ruvB*) DNA damage repair pathways of 30 s irradiated BCG Moreau and Pasteur samples.

Total RNA was extracted from the bacterial samples (as described in Section 2.2) using TRIzol (Thermo Fisher Scientific, Waltham, MA, USA) and the RiboPureTM Bacteria kit (Thermo Fisher Scientific, Waltham, MA, USA), following the manufacturer’s protocol. Briefly, bacterial cells were lysed by mechanical disruption using zirconium beads and bead-beater equipment (Biospec Products Inc., Bartlesville, OK, USA). The lysate was then subjected to chloroform extraction and the RNA-containing aqueous phase was collected for further purification using the RiboPureTM Bacteria kit. To remove any DNA contaminants, Turbo-Dnase (Ambion-Life, Waltham, MA, USA) digestion was performed according to the manufacturer’s instructions. The resulting RNA sample was then eluted in RNAse-free water and the total concentration was measured with NanoDrop 2000 (ThermoFisher Scientific, Waltham, MA, USA) equipment, and the overall RNA quality was assessed using the A260/A280 and A260/A230 ratios to detect proteins and other types of contaminants.

cDNA synthesis was performed using reverse transcriptase on 600 ng of total RNA. The synthesis was performed using random primers and the SuperScript III First-Strand Synthesis SuperMix (ThermoFisher Scientific, Waltham, MA, USA). The resulting cDNA was then used as a template for quantitative real-time polymerase chain reaction (RT-qPCR) analysis.

Specific primers (Table 1) were designed to target the genes of interest based on the genome sequence of *Mycobacterium bovis* BCG str. Moreau RDJ (Genbank accession number: AM412059): *lexA*, *recA*, *recX*, *pafB*, *pafC*, *adnA*, *adnB*, *ruvC*, *ruvA*, and *ruvB*. *sigA* was used for internal normalization. Primers were designed based on each specific gene sequence to produce an amplicon of around 100–200 bp, and for the pair Tm to be around 60 °C. Primer pair specificity was previously assessed by the presence of a single band after PCR products with Moreau and Pasteur genomic DNA agarose gel electrophoresis. PCR reactions for the RT-qPCR assays were conducted in a 7500 Real-Time PCR System (Applied Biosystem, Waltham, MA, USA) in 96-well plates under the following conditions: 95 °C/10 min (1 cycle); 95 °C/30 s, 60 °C/30 s, 72 °C/30 s (40 cycles). A dissociation curve was performed after each assay to check the quality of each primer. Gene expression levels were calculated by relative quantitation using the comparative Ct method (ΔΔCt), as previously described [28]. mRNA relative levels were normalized against sigA mRNA [29,30] and qPCR experiments were performed in triplicate. Fold-change values were calculated using BCG Pasteur expression as 1.0.

### 2.4. Statistical Analysis

Statistical analysis was performed using three independent biological samples. The survival rate after different UV exposure periods was analyzed using the one-way ANOVA test with the Dunnett post-test, using Pasteur as the control.

The RT-qPCR data were assessed for normal distribution using the Kolmogorov–Smirnov and Shapiro–Wilk tests. Significance was determined using Student’s *t*-test with a *p*-value threshold of ≤0.05.

## 3. Results

### 3.1. M. bovis BCG Moreau Is More Susceptible to UV Exposure Than BCG Pasteur

**Figure 1:**
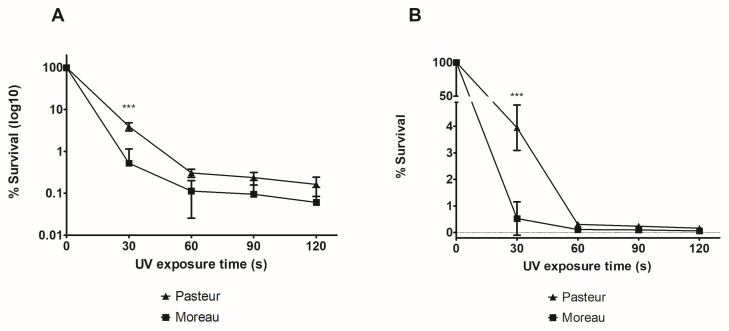
**Survival (%) profile of *M. bovis* BCG Moreau compared to BCG Pasteur.** After UV exposure of BCG Moreau and Pasteur for different periods, viability was assessed using CFU, comparing irradiated to non-irradiated cells. In (**A**), data are shown in log10 values, whereas in (**B**) absolute survival rate values are plotted. In (**B**), Y-axis segmentation was performed to better assess differences detected between Moreau and Pasteur strains. *p*-value: *** < 0.001. Data represent the mean ± SD of three independent experiments.

To standardize the UV survival assay, we tested different exposure periods, comparing the known HR efficient BCG Pasteur strain to the HR-deficient BCG Moreau. As shown in Figure 1, after 30 s exposure, BCG Pasteur shows a 25× reduction (3.94 ± 0.85%) in survival compared to the non-irradiated sample (t = 0), whereas BCG Moreau shows a 190× reduction (0.52 ± 0.63%). This difference is statistically significant, and, in longer exposure periods, we could not detect a distinct pattern between strains, a probable indication that longer UV exposure has an equal bactericidal effect on BCG cells.

### 3.2. Early BCG Strains, except Russia, Are like Moreau, Having Lower UV Exposure Survival Rates

**Figure 2:**
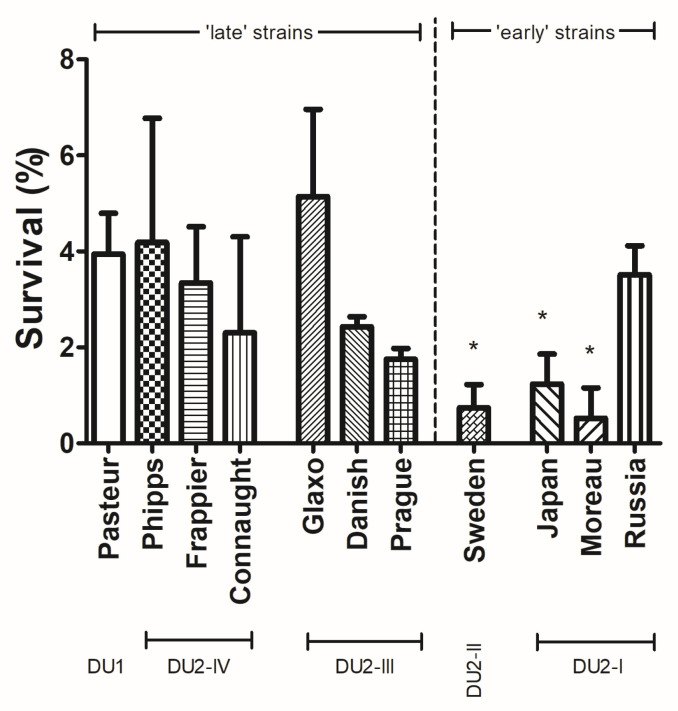
**UV exposure survival profile of *M. bovis* BCG strains representing the four different genomic groups** After 30 s UV exposure, viability (% survival) was assessed using CFU, comparing irradiated to non-irradiated cells. *p*-values: * < 0.05. Data represent the mean ± SD of three independent experiments.

After determining the best UV exposure conditions, we expanded the analysis to other members of the BCG clade in order to investigate possible evolutionary links for this HR-related phenotype. The analysis included BCG strains representing the four different genomic groups defined in [24]: DU-1 (Pasteur), DU2 Group IV (Frappier, Connaught and Phipps), DU2 Group III (Glaxo, Danish and Prague), DU2-Group II (Sweden), and DU2-Group I (Japan, Moreau and Russia). As seen in Figure 2, the early DU2-I strains, which include the HR-deficient BCG Moreau control, showed an overall statistically significant lower survival. Late strains, on the other hand, were more similar to the HR-efficient BCG Pasteur. These data indicate a tendency to connect the evolutionary story of the BCG clade to HR efficiency within this vaccine family.

### 3.3. Transcriptional Profile Reveals Lower Expression of DNA Damage Repair Pathway Genes in BCG Moreau Compared to Pasteur

**Figure 3:**
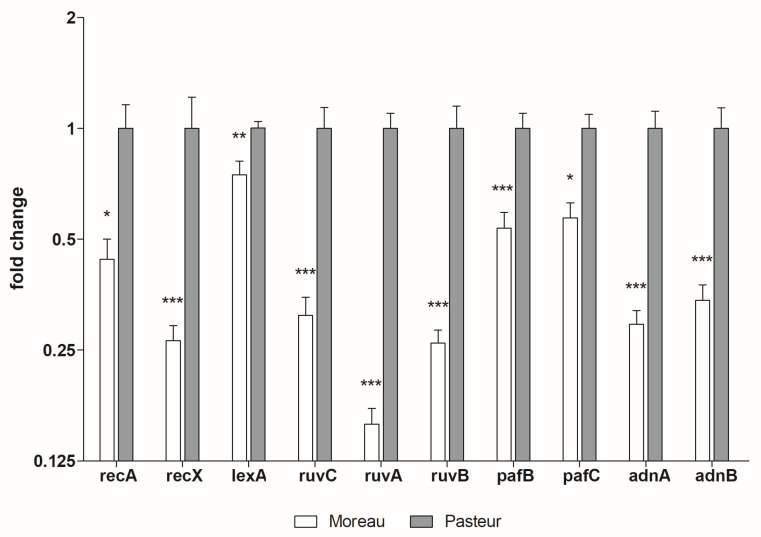
**Transcriptional profiling of the core and regulatory HR genes in *M. bovis* BCG Moreau and Pasteur**. Comparative RT-qPCR analysis with primers detecting core (*recA*, *ruvC*, *ruvA*, *ruvB*, *adnA* and *adnB*) and regulatory (*recX*, *lexA*, *pafB* and *pafC*) HR genes from BCG Moreau and Pasteur samples after 30 s UV exposure. *sigA* transcription was used as an internal normalization control. Fold change was calculated setting Pasteur values as 1.0. *p*-values: * < 0.05; ** < 0.01; *** < 0.001. Data represent the mean ± SD of three independent experiments.

To try to understand the molecular mechanisms underlying this BCG Moreau HR-deficient phenotype, we performed RT-qPCR assays to detect and quantify transcripts of the known core and regulatory HR genes described in mycobacteria. As shown in Figure 3, we observed an overall reduction in the expression of all analyzed genes for BCG Moreau when compared to BCG Pasteur. Although this transcriptomic pattern corroborates the HR-deficiency and lower UV exposure survival phenotypes, it does not fully explain them, since it does not identify the molecular agent, possibly a regulator, that leads to this low HR machinery functionality.

## 4. Discussion

*M. bovis* BCG Moreau is a known HR-deficient strain, amenable to HR-based knockout generation protocols only when external effective recombinases are added to the system [13]. In comparison, BCG Pasteur, a strain used as reference for many of the studies with BCG and an interesting surrogate model for *M.tb*, has an effective HR machinery and is able to perform all steps of DNA repair metabolism [31]. Here, we aimed to develop a protocol using BCG Moreau and Pasteur as negative and positive HR-efficiency controls to screen several other BCGs, and investigate if this HR-related phenotype is somehow linked to this clade evolution. We also addressed the molecular mechanism underlying this phenotype, determining the transcriptional profile of a set of genes known to be involved in the HR machinery in mycobacteria.

From an evolutionary perspective, deficiency in HR and other DNA repair mechanisms is a highly deleterious feature, mainly due to the introduction of mutations that lead to genome instability. In natural conditions, bacteria with such a phenotype are withdrawn from the population due to lower fitness compared to more efficient ones [27,32,33,34]. In the case of the vaccine BCG Moreau, as lower selective pressure is applied during its production, this phenotype can be perpetuated under favorable culture conditions. Furthermore, from the point of its possible use as a recombinant biopharmaceutical, HR deficiency is an advantage, providing a more stable product for the introduction and maintenance of exogenous DNA [35]. In humans, for example, HR deficiency is usually correlated with cancer [36,37], and, in some cases, it is known that a pathogen, such as *Chlamydia trachomatis*, can inhibit HR metabolism in cells from cervical and ovarian tissues, leading to carcinogenesis [38].

UV exposure is known to induce DNA damage, mainly by forming adjacent pyrimidine dimers which may lead to single- or double-strand breaks during repair, lowering the DNA replication rate and activating the SOS response (LexA/RecA dependent), a global cell response in which several pathways are modulated, such as DNA repair mechanisms [15,16,22]. HR-defective strains (in one or several pathways genes) are more susceptible to the bactericidal effect of this non-ionizing radiation, so UV exposure followed by CFU counting to estimate survival is a simple, cheap, and quick assay to assess HR efficiency. For example, this approach was used to unravel bacterial genes important for this metabolism, such as *recA* [26], *recX* [23], *recFOR* [39], *adnA*, and *adnB* [39,40], among others. This same approach also showed that RecBCD has a different role in mycobacterial DNA repair mechanisms than that already described for *Escherichia coli* [17,40].

Our results show that UV has indeed a bactericidal effect on BCG cells, as even after 30 s exposure, survival diminished about 25× for Pasteur (final % survival of 3.94 ± 0.85) and 190× for Moreau (final % survival of 0.52 ± 0.062). It is important to emphasize that the values obtained for BCG Pasteur are similar to those previously described in [26], but that Moreau’s behavior differs from the 10,000× reduction observed for the Pasteur Δ*recA* strain, corroborating that this locus is not the answer to the Moreau HR-deficient phenotype. Works with *recO* [39], *ruvA* [41], and *recX* [23] knockouts also show a higher reduction in survival compared to the wild-type strain than what is observed in our assay with BCG Moreau. This raises the hypothesis that the observed HR deficiency phenotype in BCG Moreau is not due to a complete lack of expression of some of the core and/or regulatory HR genes.

In longer UV exposure periods, almost all bacteria were dead, with a statistically equal survival rate lower than 0.5%. But, to achieve our main purpose, we set a condition (30 s exposure) which allowed us to statistically differentiate BCG Pasteur and Moreau, here used as HR-efficient and HR-deficient control strains, respectively. We then expanded our analysis to other BCG strains, and could observe that early strains behave like Moreau, except for Russia, indicating that the HR-deficient phenotype could have been a plesiomorphic feature. Regarding Russia’s phenotype, it is now known that what we call BCG Russia is indeed a mixture. For example, there is a report of a *recA^−^* Russia strain, suggesting it as the cause of its HR-deficient phenotype [42]. But, other studies do not identify this mutation and observe a HR-efficient phenotype, like that of BCG Pasteur (and as we found in our UV exposure assays). Some authors correlate this discrepancy with BCG Russia not being clonal, but rather considered as a population of related bacteria [43].

In the other evolutionary pole of late strains, bacteria have higher survival after UV exposure, indicating a more efficient HR machinery. This result indicates a correlation between the historical and genomic progression story of the in vitro BCG evolution to the more HR-efficient phenotype. With that, the deficiency would be the plesiomorphic trait, possibly present solely in the original BCG, as natural *M. bovis* has large amounts of HR events described, important for its genome evolution [44]. In some way yet to be discovered, the attenuation of the *M. bovis* clinical strain in the Pasteur Institute in Lille, under the conditions employed, may have led to this HR-deficient phenotype, restored later in the BCG clade evolution.

In mycobacteria, there are three double-strand break (DSB) DNA repair metabolisms known: the error-prone pathway via non-homologous end-joining (NHEJ) catalyzed by Ku and DNA ligase D (LigD); the error-free HR pathway; and the mechanism of single-strand annealing (SSA) [40]. Different from other bacteria such as *Escherichia coli*, in which the RecBCD complex works in the strand-end resection of HR metabolism, in mycobacteria, this complex has the same function, whereas in the SSA pathway, the complex AdnA/AdnB plays this role in mycobacterial HR metabolism [17,39,40]. On the other hand, the RecFOR complex works in the pre-synapse step of the single-strand DNA repair pathway (SSB) in mycobacteria [17,39]. So, the core enzymes for the HR DNA repair metabolism are mostly the same between bacteria (RecA loading on the derived single strand, and single strand invasion and resolution of Holliday junctions), except for the pre-synaptic step (*adnA*, *adnB* for DSB and *recF*, *recO*, and *recR* for SSB in mycobacteria and *recB*, *recC*, and *recD* for *E. coli*, for example).

Besides differences in the core HR genes, mycobacteria show additional regulatory pathways to the known RecA/LexA-dependent SOS response [16,18,22], well described for *E. coli*. In this case, LexA is a repressor acting in the promoter (P2) of the *recA*/*recX* operon, and once a single strand is formed elsewhere due to DNA damage, basal-produced RecA binds to it, leading to LexA destruction, increasing the transcription of *recA*/*recX* and other LexA-regulon genes. It is also important to note that in the intergenic region between *recA* and *recX* CDSs, there is a *recX* proper constitutive promoter [45], strong enough to make a significant contribution to the overall RecX expression level in the absence of DNA damage, as both *recA* promoter (P2 and P1 [46]) are inducible by DNA damage. Probably, this would control basal RecA activity in the absence of DNA-damaging conditions when the HR metabolism should not be active.

The second *recA* promoter (P1) is induced by the PafB/PafC complex. To act as an activator, this complex has to go through modifications that are induced by DNA damage, but the proper molecular mechanism for this activation is not yet clear [19]. So, in a DNA damage situation, both *recA* promoters are functional and increase the overall RecA expression. Besides regulating the *recA*/*recX* operon, LexA can also repress its own transcription and that of the *dnaA* and *ruvABC* genes [16,19], with this last one also being induced by the activated PafB/PafC complex, together with *uvrABC*, *recBCD*, *adnAB*, and others [19]. Therefore, regarding the known core and regulatory mycobacteria HR machinery, we chose to investigate, via RT-qPCR, the transcriptional levels of the most studied and important genes: *recA*, *recX*, *lexA*, *ruvA*, *ruvB*, *ruvC*, *pafB*, *pafC*, *adnA*, and *adnB*. Our HR transcriptional profiling showed an overall lower transcript level for all studied genes in 30 s UV-irradiated BCG Moreau when compared to Pasteur. This transcriptional phenotype is consistent with the observed HR-deficient and low-survival-after-UV-exposure phenotypes. It agrees with the observed intermediate values obtained when comparing BCG Pasteur to the real HR core gene knockouts described in the literature, but still does not identify the main actor responsible for this biological phenomenon. A question yet to be answered is whether Moreau is capable of properly inducing HR gene expression under UV exposure or if it does so poorly, having an overall lower expression that even with UV induction is not comparable to BCG Pasteur.

In order to identify possible other genes that may explain this BCG Moreau HR phenotype, we performed a preliminary STRING (https://string-db.org/, accessed on 1 May 2023) analysis using these RT-qPCR genes as queries, and *M.tb* as the selected organism. From that, we retrieved other genes that are connected to the queries: *dop*; *rv0435c*; *dnaN*; *rv2191*; *rv0004*; *dinX*; *rv3644c*; *dinG*; *dinP*; *rv2413c*; *rv1251c*; *rv1278*; *rv0434*; *rv1277*; *rv2735c*; *topA*; *rv3649*; *uvrD1*; *uvrD2*; *helZ*; *polA*; *rv2529*; *rpoC*; *xthA*; *dnaE2*; *dnaE1*; *dnaQ*; *dnaZX*; *recF*; and *pafA*. The alignment of the coding sequence (CDS) plus the 200 bp upstream of the orthologous genes from BCG Moreau and Pasteur shows 100% identity, except in the case of *rpoC*, in which a single nucleotide change in the CDS alters the protein sequence, making Moreau RpoC different from its homologs in *M.tb* H37Rv and BCG Pasteur and Tokyo by 1aminoacid, A83T. This gene codes for the RNA polymerase beta subunit and, together with *recA*, has been shown to be downregulated in *M. smegmatis* during nitric oxide exposure [47]. Furthermore, recent work shows that the PafBC activated complex induces its regulon expression by structurally readapting the sigma factor within the RNA polymerase complex. In this case, the whole complex can interact with a specific −26 element present within the promoter region of all PafBC-regulated genes, additional to the usual −10 element. In this way, under DNA damage, PafBC adapts the sigma factor to recognize the specific −26 element, leading to the transcription of PafBC-dependent genes [48]. Thus, one can speculate that a *rpoC* mutation could interfere with the induction of the HR machinery under DNA damage conditions. Although this is an interesting finding, future work will be performed to check whether this hypothesis can explain the BCG Moreau HR-deficient phenotype. Nonetheless, it is important to emphasize that this *rpoC* mutation is exclusive to BCG Moreau and would not explain the low UV exposure survival observed for the remaining early strains.

It is long known that some mycobacteria, especially those that are important human and animal pathogens, are very genomically stable regarding lateral gene transfer, and this is due to several reasons, such as low growth rate and cell wall structure, among others [43]. This contrasts with other human pathogens that are able to acquire plasmids, genetic mobile elements, and DNA fragments from the environment carrying pathogenicity, virulence, and antibiotic-resistance genes, which can improve bacterial fitness [49,50]. This acquired genetic information can be added to the main genome via several molecular mechanisms, including HR metabolism [51,52], which can also be used, as previously discussed, as a DNA repair mechanism. For mycobacteria, evolution is mainly due to mutations in its genome, represented by deletions, insertions, or single nucleotide polymorphisms (SNP) [53]. For example, acquired resistance towards anti-tubercular compounds has been shown to occur due to a single SNP in drug target genes, including *rpoB* [54] and *katG* [55].

In this case, HR metabolism usage in the natural bacteria life cycle and in the research laboratory environment is widely different regarding mycobacteria. In the lab, HR-based methodology can be used to create knockout strains, thus altering the mycobacteria genome. In nature, HR is mainly used in the opposite way to maintain the stability of the mycobacterial genome, and the regulation of these pathways is directly connected to the infection success of pathogenic mycobacteria [53]. Therefore, an improved understanding of the role of DNA repair in the pathogenesis of mycobacteria might reveal good candidate targets for new effective treatments against tuberculosis. There is much data regarding the expression of each DNA repair gene in *M.tb*; however, as we show in this case for BCG, other still unknown genes may be involved in HR metabolism. Additional omics assays will be performed comparing UV-induced and non-induced BCG Moreau and Pasteur cells to further identify the responsible locus for this differential phenotype, and to determine if Moreau does in fact induce its HR genes under UV exposure. Unraveling the reason for this discrepant HR phenotype between BCG Moreau and Pasteur may lead to the discovery of a new HR regulator, a possible important target for the development of strategies to treat tuberculosis.

We show that even with a simple UV irradiation survival rate assay, it is possible to detect the discrepant HR machinery functionality between BCG Moreau and Pasteur strains, and that this phenotype is broadened to early and late strains within the BCG evolution clade. Notwithstanding, further assays should be performed to identify the molecular agent responsible for the overall reduction in HR core and regulatory gene expression found in BCG Moreau. As the genes analyzed in this work are the ones described to undergo this metabolism in the mycobacteria literature, our RT-qPCR was based on them; however, others that are yet to be discovered must also be involved and need to be identified and further studied. This could contribute to TB drug development research, as inhibiting this gene product could impair *M.tb*. DNA repair during the infectious process, leading to a less virulent pathogen more easily cleared by immune cells.

## Figures and Tables

**Table 1:** Primer list for RT-qPCR assay.

Primer Name		Sequence (5′-3′)
*Locus*	Direction	
*lexA*	For	CTTACCGGAACCCACCTTTG
	Rev	GCTTCGACCATCGAGTCAC
*recA*	For	CTGAATAATTCGGGCACCAC
	Rev	GCGTTGGTACCGTCCTTGA
*recX*	For	CTGGTGGATGACACCGACT
	Rev	GTACCAGCTTTTCCGCCCG
*pafB*	For	ACAAGAACGAGCTGCGTGAC
	Rev	ATCCGGGGTCAGCTCGACA
*pafC*	For	GAACAGGCACCCACAGAAAG
	Rev	ATCAACAGCACCCGGATGG
*adnA*	For	GACCGCCTTGTTCGACATC
	Rev	GGCATGTGCGCTAAGGAC
*adnB*	For	CTGCTGGCTTATTTGGACGT
	Rev	CACCACCTGCCATTCCAAG
*ruvC*	For	GAGGTGGTGGCTATCGAAC
	Rev	TGAGCCTTGTCTGCGGAAC
*ruvA*	For	GAACGCATGGTGTTGGAACT
	Rev	TGTTTGGCCGCAAAGCCCA
*ruvB*	For	ATGGAAGACTTCCGCGTCGA
	Rev	GTAGAAATCCATGTGCGCGG
*sigA*	For	CTCGACGCTGAACCAGACT
	Rev	AGGTCTTCGTGGTCTTCGC

## Data Availability

The data presented in this study are available in the article.
